# Effect of Four Different Initial Drying Temperatures on Biochemical Profile and Volatilome of Black Tea

**DOI:** 10.3390/metabo15020074

**Published:** 2025-01-25

**Authors:** Zaifa Shu, Huijuan Zhou, Limin Chen, Yuhua Wang, Qingyong Ji, Weizhong He

**Affiliations:** 1 Lishui Institute of Agriculture and Forestry Sciences, Lishui 323000, China; shuzaifa@163.com (Z.S.); lsszhj1020@163.com (H.Z.); clmit@zju.edu.cn (L.C.); 2 College of Horticulture, Nanjing Agricultural University, Nanjing 210095, China

**Keywords:** catechins, drying temperature, black tea, sensory evaluation, tea processing, volatilome, theaflavins

## Abstract

Background: Black tea processing conditions significantly affect the final taste and flavor profiles, so researchers are now focusing on developing equipment and improving the appropriate processing conditions of major black tea varieties. Methods: Here, we tested the effect of four different initial drying temperatures, i.e., R65 (65 °C), R85 (85 °C), R105 (105 °C), and R125 (125 °C), on the sensory and biochemical profiles and volatilome of the black tea variety “Lishui wild” (LWV). Results: Our results indicate that both 85 and 105 °C are better than 65 and 125 °C for initial drying for 20 min. R105 had the highest sensory evaluation scores due to better shape, aroma, taste, leaf base, thearubigins, theanine, caffeine, and ratio of theaflavins + thearubigins to theaflavins. Both R85 and R105 had higher catechins than R65 and R125. The LWV volatilome consisted of esters (19.89%), terpenoids (18.95%), ketones (11.3%), heterocyclic compounds (9.99%), and alcohols (8.59%). In general, acids, aldehydes, amines, aromatics, ethers, hydrocarbons, phenols, sulfur compounds, and terpenoids accumulated in higher amounts in R85 and R105. The highly accumulated compounds in R105 were associated with green, fruity, sweet, woody, floral, hawthorn, mild, nutty, powdery, rose, and rosy flavors. The main pathways affected are secondary metabolites, sesquiterpenoid and triterpenoid biosynthesis, glycerolipid metabolism, zeatin biosynthesis, phenylpropanoid biosynthesis, ABC transport, glutathione metabolism, etc. Therefore, R105 can be used to achieve the optimal taste, flavor, and aroma of LWV. Conclusions: Overall, the presented data can be used by the tea industry for processing black tea with the most optimum volatile substances, catechins, theanine, amino acids, and other compounds.

## 1. Introduction

Black tea (*Camellia sinensis*), one of the six tea types, originated in China and then spread to the world. As of now, it is the most consumed beverage in the world [1]. China produces ~50.6% of the global tea, followed by India, Kenya, Turkey, Sri Lanka, and others. The major tea-producing regions in China are Anhui, Zhejiang, Fujian, Shaanxi, Hubei, Hunan, Sichuan, Guizhou, and Yunnan [2]. Black tea contains a range of health-beneficial compounds such as amino acids, catechins, polyphenols, pigments (theaflavins, thearubigins, and theabrownins), polysaccharides, alkaloids, and saponins. These compounds have health-beneficial properties such as antioxidant, anti-inflammatory, anticancer, anti-diabetic, anti-obesity, and anti-microbial activities. Moreover, they are also associated with immune regulation and cardiovascular, gastrointestinal, reno-, and hepato-protection [3].

Black tea is processed either by the orthodox method or by the crush, tear, and curl method. The first method includes withering, maceration, fermentation, and drying. The processing of black tea can change the composition and content of tea metabolites [4]. During withering, tea leaves are spread out in a withering system (chemical or physical) to minimize the moisture content. Next, the withered tea is macerated (or rolled). This is carried out to rupture cell structures and allow the major bioactive compounds to react during the next stage. In the fermentation stage, the most important reactions happen which are ultimately associated with the tea quality. The fermentation time, temperature, oxygen, and relative humidity influence the quality of the tea [5]. Finally, the fermented tea leaves are dried to stop the fermentation process. This stage ensures that the oxidation and enzymatic processes are stopped and that moisture is at the desired level (3–4%), so that the handling and transportation are easy [4,6]. This step is very critical for black tea processing since the reduction in moisture content and inhibition of the fermentation process improve the sensory quality of the tea including flavor, aroma, soup color, and taste [7]. Changes in the temperature during drying have been associated with the biochemical processes and their inhibition. It has been shown that the enzymatic activities are temperature-dependent and can cause changes in chlorophyll, polyphenols, catechins, and other flavoring compounds [6,8]. Drying is carried out at two stages, i.e., initial heating at suitable spread thickness, air flow, and drying time followed by a second drying to limit the moisture content in the final product. Several studies have tested the effect of drying temperature on biochemical profile and volatilome and reported that 96 °C [9], 100 °C [8], 110 °C, and 120 °C [7] can affect these characteristics. The key changes during the drying include changes in carboxylic acid, Maillard reactions, caramelization [10], reduction in volatile compounds, and others [11].

Currently, researchers and industrialists involved in tea processing are focusing on developing equipment, comparing drying methods, and finding the most effective temperature for processing major tea varieties [12,13]. In our ongoing research, we are focusing on the Gonfu black tea processing technology in Lishui, China. In this regard, in this research, we have tested how different drying temperatures (initial drying stage) affect the sensory, biochemical (amino acid and catechin), and volatilome profiles of the black tea variety “Lishui wild”. Here, we tested four initial drying temperatures, i.e., 65 °C (R65), 85 °C (R85), 105 °C (R105), and 125 °C (R125). We evaluated the sensory profiles of the teas, including leaf shape, color, aroma, flavor, leaf base (leaf bottom), pigment (theaflavins, thearubigins, and theabrownins), amino acid content, and volatiles. Our results indicate that temperature is a critical factor during black tea drying and can significantly influence quality characteristics. This study provides a basis for the application of black tea processing technology for initial drying in the industry.

## 2. Material and Methods

### 2.1. Plant Material and Processing Method

Lishui wild variety (*Camellia sinensis*) (LWV) was used as the plant material. The tea plants are grown in Lishui, Zhejiang, China according to standard agronomic practices. Tea leaf samples (one bud and two leaves) were collected on 26 May 2024 and processed according to the standard method of black tea, i.e., withering, rolling, fermenting, and drying. In total, 50 kg of tea leaves were withered and kept at a room temperature of 27 °C and indoor relative humidity of 68%~70%. Fresh leaves were placed on a drying rack with a thickness of 5 cm and withered for 12 h until the moisture content reached 60%~62%. The leaves were then kneaded in a Chunjiang 6CR-Z55 tea rolling machine (Zhejiang Chunjiang Tea Machinery Co., Ltd., Hangzhou, China) for 70 min (25 min empty kneading, 15 min light kneading, 10 min heavy kneading, and 20 min gentle kneading), followed by fermentation for 3 h (temperature inside the fermentation chamber is 35 °C, relative humidity is 95%). Light and heavy kneading correspond to light and hard pressure of the rolling barrel on the leaves, respectively. Four treatments (R65, initial drying at 65 °C; R85, initial drying at 85 °C; R105, initial drying at 105 °C; and R125, initial drying at 125 °C) were set for the initial firing, with four replicates. The first firing time was 20 min, and the leaf thickness was 2 cm. Finally, the leaves were cooled for 20 min and reheated to 80 °C for 60 min, after which the leaves were spread to a thickness of 4 cm. Samples were collected and analyzed in four replicates for each of the four treatments.

### 2.2. Sensory Evaluation, Water Extract, Catechins, Amino Acid, and Total Phenol Content Determination

Sensory evaluation and caffeine content quantification of the processed tea and/or soups was carried out according to the Chinese national standard procedure GB/T 23776-2018 [14]. According to the national standard GB/T 23776-2018, the soaking conditions are 100 °C for 5 min. Water extracts of the triplicate samples were determined according to standard method GB/T 8305-2013 of the National Standards of People’s Republic of China [15]. Amino acid contents were determined according to standard method GB/T 8314-2013 of the National Standards of People’s Republic of China [16].

Catechins were determined according to standard method GB/T 8313-2018 of the National Standards of People’s Republic of China [17]. For the determination of catechins, 0.01 g of ground tea leaf sample was taken for each replicate, 1 mL of preheated 70% methanol was added, homogenized, extracted at 70 °C for 0.5 h, and centrifuged at 12,000 rpm for 5 min, and the supernatant was filtered for analysis on the UltiMate 3000 (ThermoFisher, Waltham, MA, USA). The mixed samples for the treatment material extracts were used as standard. For amino acid content determination, 0.5 g of the evenly mixed samples were ground and 5 mL of the extract was mixed with 1 mol/L of hydrochloric acid solution at room temperature by ultrasonic oscillation for 60 min and kept for 1 h.

The experimental conditions were as follows: chromatography column: Hypersil GOLD 250 mm × 4.6 mm; 5 μm (ThermoFisher, Waltham, MA, USA) operating at 35 °C; injection volume: 5 μL; detection wavelength: 203 nm; flow rate: 0.8 mL/min; elution method: gradient elution (Table 1). Standard curves were plotted based on the concentrations of the standards and area.

The total phenol content (TPC) was determined using a total phenols test kit (Norminkoda Biotechnology Co., Ltd., Wuhan, China). Theanine content was determined by the Chinese national standard method GB/T 23193-2017 [18].

### 2.3. Volatilome Detection and Analysis by GC-MS

Five samples of each of the four treatments were ground to a powder, and 500 mg of the powder was immediately transferred to a 20 mL headspace vial (Agilent, Palo Alto, CA, USA) containing saturated NaCl solution, sealed with crimp-top caps with TFE silicone headspace septa (Agilent). At the time of SPME analysis, each vial was placed at 60 °C for 5 min, and then a 120 µm DVB/CWR/PDMS fiber (Agilent) was exposed to the sample headspace at 60 °C for 15 min.

After sampling, desorption of volatile organic compounds (VOCs) from the fiber coating was performed in the injection port of the GC apparatus (Model 8890; Agilent) at 250 °C for 5 min in the splitless mode. The identification and quantification of VOCs were performed using an Agilent Model 8890 GC and a 7000D mass spectrometer (Agilent) equipped with a 30 m × 0.25 mm × 0.25 μm DB-5MS (5% phenyl-polymethylsiloxane) capillary column. Helium was used as the carrier gas at a linear velocity of 1.2 mL/min. The injector temperature was maintained at 250 °C. The oven temperature was programmed from 40 °C (3.5 min), increasing at 10 °C/min to 100 °C, at 7 °C/min to 180 °C, at 25 °C/min to 280 °C, and held for 5 min. Mass spectra were recorded in electron impact (EI) ionization mode at 70 eV. The quadrupole mass detector, ion source, and transfer line temperatures were set at 150, 230, and 280 °C, respectively. The ion monitoring (SIM) mode of the MS was selected for the identification and quantification of the analytes.

### 2.4. Data Analysis

Statistical analysis methods, including t-tests and one-way analysis of variance (ANOVA), were performed using SPSS version 20.0 (SPSS, Inc., Chicago, IL, USA). *p* < 0.05 and *p* < 0.01 were considered significant differences and extreme differences.

The GC-MS data were analyzed as follows. Principal component analysis (PCA) and heatmap analysis for metabolites were performed online (https://www.metaboanalyst.ca/). Hierarchical cluster analysis and Pearson’s correlation coefficient were performed using the prcomp, cor, and ComplexHeatmap statistical functions, respectively, in R (www.r-project.org). Differentially accumulated metabolites (DAMs) were determined by variable importance projection (VIP) > 1 and absolute Log_2_FC (|Log_2_FC| ≥ 1.0). VIP values were extracted from the OPLS-DA results, which also included score plots and permutation plots, generated using the R package MetaboAnalystR version 4.0. The data were log-transformed (log_2_) and mean-centered before OPLS-DA. A permutation test (200 permutations) was performed to avoid overfitting. The compounds were annotated using KEGG Compound database [19], and the annotated metabolites were mapped to KEGG pathways [20].

## 3. Results

### 3.1. Sensory Evaluation

**Figure 1:**
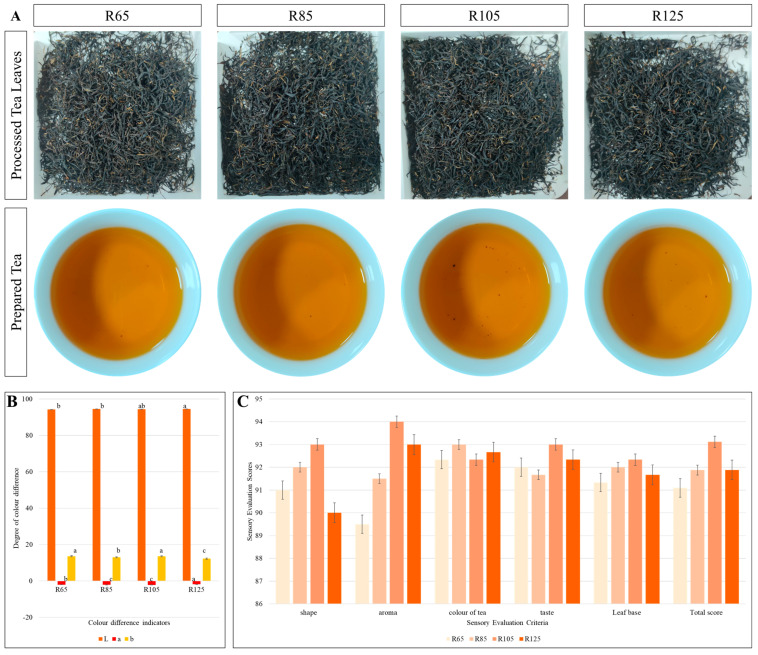
(**A**) Sensory evaluation of processed tea leaves and soup. (**B**) Sensory evaluation scores for tea leaves and soup. (**C**) Degree of color differences. R65, R85, R105, and R125 refer to processing temperatures 65 °C, 85 °C, 105 °C, and 125 °C, respectively. The graphs show mean values of four replicates. Different letters above bar graphs mean significant difference at *p* < 0.05.

**Figure 2:**
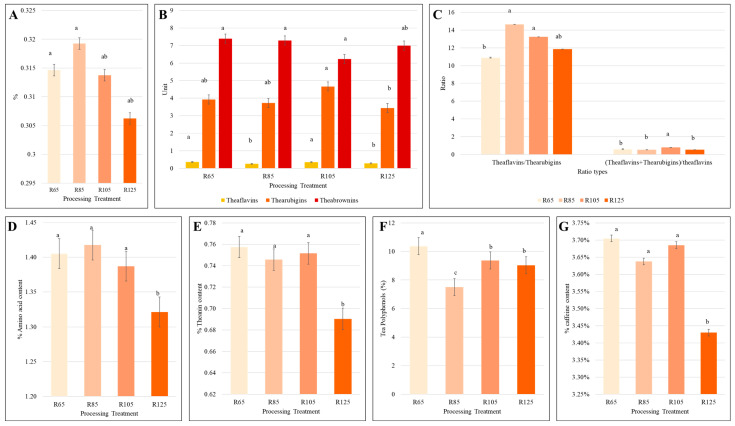
Effect of processing temperature on biochemical profile of black tea. (**A**) Water extract (%), (**B**) pigment contents (theaflavin, thearubigins, and theabrownins), (**C**) ratios of the pigments, (**D**) amino acid, (**E**) theanine, (**F**) total polyphenols, and (**G**) % caffeine content in LWV black tea processed at different temperatures. R65, R85, R105, and R125 refer to processing temperatures 65 °C, 85 °C, 105 °C, and 125 °C, respectively. The graphs show mean values of four replicates. Different letters above bar graphs mean significant difference at *p* < 0.05.

The infusions of LWV processed at four different temperatures were evaluated for color difference values (L, a, and b) (Figure 1A,B). The degree of discoloration (L) was not significant among the four treatments. The degree of the soup yellowness (+) (b) was highest for R65 and R105, followed by R85 and R125. R105 had the highest total sensory evaluation score (93.1), followed by R85, R125, and R65. This trend was based on leaf shape, aroma, leaf as well as soup color, soup taste, and leaf base (Figure 1C). The water content of the processed teas showed a decreasing trend with increasing temperature, i.e., R85 < R105 < R125. R65 and R105 had a non-significant difference in water content (Figure 2A). The observed least differences among the treatments for water content are understandable since the tea leaves had already undergone processing stages, i.e., withering, rolling, and fermentation. The processing temperature affected the color-related pigments of the tea source, i.e., theaflavins, thearubigins, and theabrownins. However, there was no trend with increasing processing temperature. R65 had the highest theaflavin content followed by R105, R125, and R85. Interestingly, R105 had significantly higher thearubigins content than other temperatures. Theaflavins content decreased from R65 to R105, but in R125 it increased again to almost the level of R85 (Figure 2B). The ratios of theaflavins to thearubigins were highest in R85 and lowest in R65. The ratios of theaflavins + thearubigins to theaflavins were highest in R105 and lowest in R25 (Figure 2C).

### 3.2. Effect of Processing Temperature on Amino Acids, Catechins, Polyphenols, and Caffeine Contents

The amino acid content was highest in R85, followed by R65, R105, and R125 (Figure 2D). There were no significant differences in theanine content in R65, R85, and R105. However, tea processed at 125 °C showed a reduction in theanine content (Figure 2E). Polyphenol content was highest at 65 °C (R65), decreased in R85, and then slightly increased in R105 and R125. The caffeine content was also highest at R65 (Figure 2F). However, it decreased slightly but not significantly in R85 and R105. R125 had the significantly lowest caffeine content. Finally, caffeine content remained highest in R65, followed by R105, R85, and R125 (Figure 2G).

Gallic acid (GA) content increased with increasing processing temperature. Gallocatechin (GC) content was highest in R85, slightly lower in R65 and R105, and lowest in R125. Epigallocatechin (EGC) content was highest in R105, followed by R85, R105, and R65. Catechin (C) content was highest in R85, followed by R65, R105, and R125. The caffeine (CAF) content was highest in R65 and R105, followed by R85 and R125. R105 had the highest epicatechin (EC) content, followed by R85, R125, and R65. Epigallocatechin gallate (EGCG) content was highest in R105, followed by R85 and R65, whereas R125 had the minimum EGCG. Regarding gallocatechin gallate (GCG), R85 had the highest content followed by R65, R105, and R125. Epicatechin gallate (ECG) content was highest in R105, while other processing treatments did not have significantly similar contents. Finally, catechin gallate (CG) content was highest in R85, followed by R105, R65, and R125 (Table 2). These observations indicate that both R65 and R125 are not suitable processing temperatures. On the other hand, the optimum processing temperatures are either R85 or R105.

### 3.3. Global Volatilome Profiles of LWV Processed at Different Temperatures

**Figure 3:**
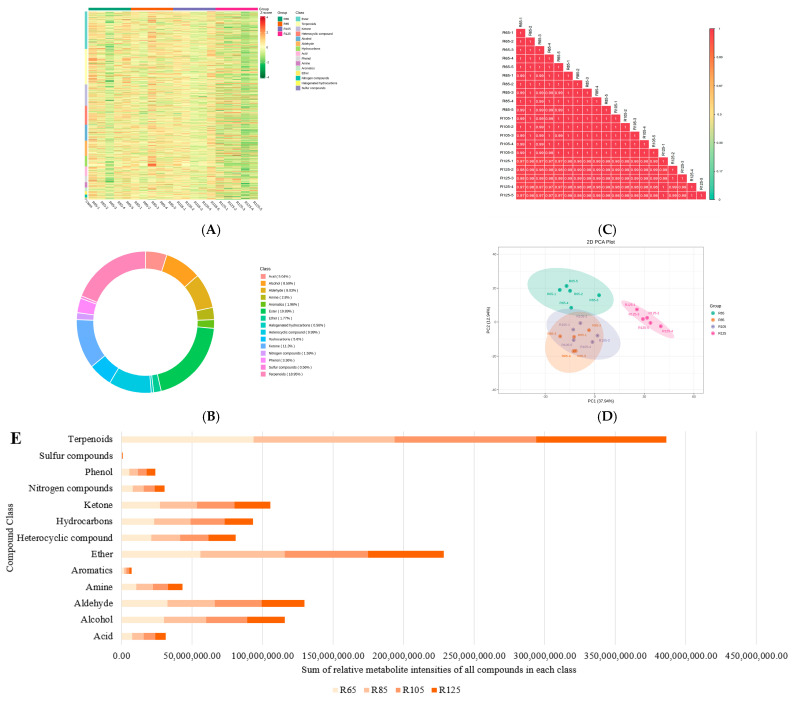
Global volatilome profile of LWV tea processed at different temperatures. (**A**) Heatmap of the relative metabolite intensities of the detected compounds in each class. (**B**) Proportion of the detected compounds in each class. (**C**) Pearson’s correlation and (**D**) principal component analysis between treatment replicates. (**E**) R65, R85, R105, and R125 refer to processing temperatures 65 °C, 85 °C, 105 °C, and 125 °C, respectively. The numbers 1–5 with each treatment indicate the replicates.

GC-MS analysis of 20 LWV samples revealed 1072 metabolites belonging to 15 compound classes (Figure 3A). The highest proportion of compounds belonged to esters (19.89%), followed by terpenoids (18.95%), ketones (11.3%), heterocyclic compounds (9.99%), and alcohols (8.59%) (Figure 3B). There was a higher correlation between replicates, indicating the reliability of the sampling (Figure 3C). This was further evident in the PCA analysis, where the replicates for each treatment were grouped closer together. A notable result was that the R105 and R85 replicates were grouped together (Figure 3D). This trend was also visible in the sensory and biochemical profiles of the four treatments, where most characteristics were similar between the two treatments (Figure 1 and Figure 2). Among the compound classes, the highest sum of relative metabolite intensity was observed for terpenoids, followed by ethers, aldehydes, alcohols, and others. In general, acids, aldehydes, amines, aromatics, ethers, hydrocarbons, phenols, sulfur compounds, and terpenoids accumulated in higher amounts in R85 and R105. In contrast, alcohols, heterocyclic compounds, ketones, and nitrogen compounds decreased with temperature (Figure 3E).

### 3.4. Differential Volatilome Profiles of LWV Processed at Different Temperatures

**Figure 4:**
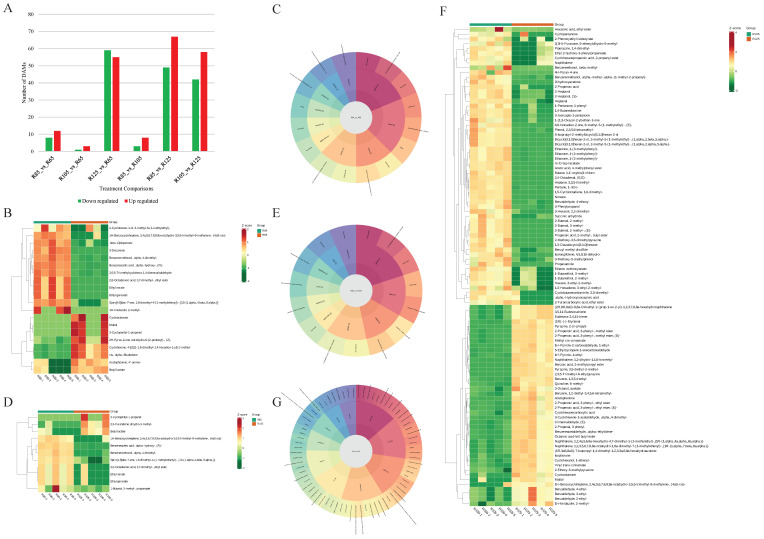
Differential volatilome profile of LWV processed at different temperatures. (**A**) Number of differentially accumulated metabolites. (**B**) Heatmap of the relative metabolite intensities and (**C**) flavor profiles of DAMs between R65 and R85. (**D**) Heatmap of the relative metabolite intensities and (**E**) flavor profiles of DAMs between R85 and R105. (**F**) Heatmap of the relative metabolite intensities and (**G**) flavor profiles of DAMs between R105 and R125.

Among the four treatments, the number of significantly differentially accumulated metabolites (DAMs) ranged from 4 (R65 vs. R105) to 116 (R85 vs. R125). In general, the number of up-regulated compounds increased from lower to higher temperatures, except for the R65 vs. R125 comparison (Figure 4A). R65 vs. R85 resulted in increased accumulation of acid (benzeneacetic acid), alcohol (benzenemethanol), esters (ethyl nerate, ethyl geranate, and 2,6-octadienoic acid, 3,7-dimethyl, ethyl ester), 1H-imidazol, 2-methyl, ketone (3-decanone), and several terpenoids. These compounds were associated with green, sweet, woody, banana, floral, hawthorn, herbal, mild, nutty, powdery, and rosy flavors (Figure 4B,C). On the contrary, 3-cyclopentyl-1-propanol, butyl lactate, maltol, 2H-pyran-2-one, tetrahydro-6-(2-pentenyl)-, (Z)-, cyclooctenone, and some terpenoids decreased in R85 compared to R65. Their flavor profiles were green, caramel, and creamy (Figure 4B,C). The metabolites were enriched in the biosynthesis of secondary metabolites and the sesquiterpenoid and triterpenoid biosynthetic pathways (Figure S1).

When R85 was compared with R105, two esters (ethyl nerate and 1-butanol, 3-methyl-, propanoate), terpenoid (spiro[4.5]dec-7-ene, 1,8-dimethyl-4-(1-methylethenyl)-, [1S-(1.alpha.,4.beta.,5.alpha.)]-), alcohol (benzenemethanol, .alpha.,4-dimethyl-), acid (benzeneacetic acid), and esters (ethyl geranate and 2,6-octadienoic acid, 3,7-dimethyl-, ethyl ester) were increased (Figure S2). The compounds were mostly associated with green, fruity, sweet, woody, floral, hawthorn, mild, nutty, powdery, rose, and rosy flavors. On the other hand, the content of butyl lactate, 2,5-furandione, dihydro-3-methyl-, and 3-cyclopentyl-1-propanol decreased in R105 compared to R85 (Figure 4D,E). When comparing R85 with R125, 126 metabolites were differentially accumulated. In general, acids, alcohols, ketones, and nitrogen compounds were higher in R125 than in R85. The most accumulated compounds in R125 were amine (propanamide), terpenoids, ketone (e.g., 3-mercapto-2-pentanone), heterocyclic compounds (e.g., 2-methoxy-3,5-dimethylpyrazine), etc. In contrast, the most abundant metabolites in R85 included pyrazine, 3,5-diethyl-2-methyl-, 2,3,5-trimethyl-6-ethylpyrazine, isophorone, cyclooctenone, benzoic acid, 2-methylpropyl ester, and others (Figure S3). These compounds were enriched in glycerolipid metabolism, zeatin biosynthesis, phenylpropanoid biosynthesis, etc. (Figure S4).

Next, the comparison of R105 with R125 revealed the maximum number of DAMs. The most abundant compounds in R125 compared to R105 were 3-mercapto-2-pentanone, propenamide, dihydroxyacetone, succinic anhydride, 1-(1,3-oxazol-2-yl)ethan-1-one, propanoic acid, and others, whereas the compounds with the highest content in R105 were 2,3,5-trimethyl-6-ethylpyrazine, pyrazine, 3,5-diethyl-2-methyl, benzoic acid, isophorone, and others (Figure S5). The DAMs between R105 and R125 were associated with nutty, almond, pungent, green, woody, fruity, sweet, spicy, and balsamic flavors (Figure 4F,G). These compounds were enriched in glycerolipid metabolism, zeatin biosynthesis, phenylpropanoid biosynthesis, ABC transport, glutathione metabolism, and others (Figure S6). These are almost similar pathways in which the DAMs of R85 vs. R125 were enriched.

To understand the overall trend of compounds in the treatments, we combined all common DAMs in all possible comparisons. Acids showed a mixed trend. Alcohol content was higher in R125 compared to both R85 as well as R125. They showed a mixed accumulation pattern from R85 to R125 as well as from R105 to R125. Amines decreased from R65 to R125 but increased in R125 more than in R85 and R105. Aromatics increased from R65 to R125 but decreased in R125 compared to R85 and R105. Esters showed mixed accumulation from lower to higher temperatures. Ethers decreased from R65 to R125 but increased in R125 compared to R85 and R105. Heterocyclic compounds showed mixed trends. Hydrocarbons, nitrogen compounds, and ketones decreased from R65 to R125 but increased from R85 to R105. Terpenoids mostly increased from R65 to R85 and R125 but decreased from R85 and R105 to R125 (Supplementary Table S1). These results generally confirm that R85 and R105 are better in terms of their volatilome profiles and contents.

Our results suggest that processing temperature affects not only the sensory quality of LWV but also the amino acid content, catechin content, and metabolites belonging to 15 compound classes. Major pathways affected by processing temperature include secondary metabolites, sesquiterpenoid and triterpenoid biosynthesis, glycerolipid metabolism, zeatin biosynthesis, phenylpropanoid biosynthesis, ABC transport, glutathione metabolism, and others. Overall, the sensory, biochemical, and volatilome profiles indicate that R85 and R105 are optimal temperatures for processing LWV black tea.

## 4. Discussion

Innovative technologies are required to maintain a balanced level of healthful components in black tea. A major focus of recent technological improvements includes improved extraction methods, enzymatic processing, fermentation, co-fermentation, chemical protection of major bioactive compounds, and temperatures during tea processing [21]. In this regard, we tested four different initial drying temperatures during the processing of the black tea variety LWV. Previous research has shown that 60–140 °C is usually applied to stop the enzymatic activities [6,10]. For effective drying and to achieve optimum yield of volatile and health-promoting compounds, an initial drying for a short time, cooling, followed by a longer drying are carried out. For example, an experiment on heat exposure from 70 °C to 140 °C for 1–64 min showed that 90, 100, and 110 °C for 16 min obtained the highest sensory scores [6]. In this regard, our initial drying time of 20 min is relevant. Moreover, the results showing that 105 °C had the highest sensory evaluation scores are consistent with this finding. This is due to the highest scores for shape, aroma, taste, and leaf base (Figure 1). It should be noted that low drying temperature (65 °C, as in our experiment) received the lowest overall sensory evaluation scores. Previous research has shown that lower drying temperatures do not effectively stop fermentation [22]. This is evident from the lower color, flavor, and aroma scores at 65 °C (Figure 1). The higher sensory scores for R105 are also consistent with theaflavin and thearubigin content and theaflavin + thearubigin/theaflavin ratio. These pigments not only affect the color and gloss of the soup but also, to some extent, the flavor and taste [23]. Although lower temperature does not stop the fermentation completely, it still has some positive effects on the tea as noticed in the case of higher theanine, polyphenol, and caffeine contents (Figure 2), consistent with previous research [10]. Other than the theaflavins and amino acids, caffeine is one of the most important compounds in tea owing to its utility in pharmaceuticals [24]. Its content is higher in fermented tea and is linked with the disintegration of caffeine–theaflavin aggregates [25]. This could be the reason why we observed the highest caffeine content in R65 [26]. Since both R65 and R105 had higher theaflavins, the higher caffeine contents at both temperatures are understandable (Figure 2C,G). It is usual that higher temperatures can lead to reduced caffeine content [6]. Thus, the optimum temperature for LWV processing should be 105 °C.

Catechins, a class of phenolic compounds, are one of the most important bioactive compounds found in teas. Their content is affected by temperature changes during tea processing [27]. In LWV, like many other teas, the EGCG and GCG accounted for the largest catechin fractions [28]. Generally, the catechins, when subjected to higher temperatures, are thermally degraded [29], as indicated by the increase in GA content with drying temperature (Table 2). Moreover, our results are consistent with this, as GC, C, EC, EGCG, ECG, and CG contents, under a higher temperature of 125 °C, showed reduction (Table 2). Previous research has indicated that higher temperatures thermally degrade tea polyphenols during processing isomerization [10,30]. Overall, considering the sensory evaluation, theaflavins, and its ratios with thearubigins, amino acid, theanine, total polyphenols, caffeine content, and catechins content, R105 proved to be a suitable initial drying temperature, followed by R85.

Aroma and flavor are the key tea characteristics that influence consumer choice. In addition to the differences between tea varieties, processing conditions also have a significant impact on volatilome profiles [31,32]. In order to achieve the optimal composition and levels of flavor, aroma, and other health-promoting compounds, it is important to optimize the processing technology. Each processing step affects the volatilome differently [33,34]. Our data are very important in this regard, especially because of the lack of studies on the effect of initial drying temperatures during tea processing. Other research has mainly explored the effects of moisture content [13], drying methods [35], secondary drying [36], fermentation [37], and others on tea quality. Our results indicate that acids, aldehydes, amines, aromatics, ethers, hydrocarbons, phenols, sulfur compounds, and terpenoids accumulated in higher amounts in R85 and R105 (Figure 3), which is consistent with the sensory evaluation (Figure 1 and Figure 2). In addition, the increase in compounds associated with green, sweet, floral, herbal, nutty, rosy, and banana flavors from R65 and R85 to R105 is consistent with the highest sensory scores of R105 (Figure 1). The fact that many DAMs between R105 and R125 were associated with pungent, green, woody, fruity, sweet, spicy, and balsamic flavors indicates that the highest temperature affects the flavor of LWV. These observations are consistent with previous research indicating that heat processing can significantly affect the flavor profiles of tea infusions [38]. Thus, our results indicate that the highest temperature affects the content of a large number of metabolites (Figure 4). Earlier research on the drying conditions of teas has revealed that temperatures such as 110 °C for 20 min are good for achieving good sensory profiles [39], which is consistent with our results that R105 has good sensory profiles of LWV. Therefore, it can be used for achieving the optimum taste, flavor, aroma, and content of health-beneficial compound classes. However, before the application of the studied parameters on the related black tea varieties, it is important to optimize and evaluate the effect of these conditions. Identification of the changes in biochemical profiles, metabolome, and volatilome of the related black tea varieties will be needed.

## 5. Conclusions

Recent research has focused on improving tea processing technology to achieve the best sensory profiles of black tea. We tested four different temperatures for the initial drying of LWV black tea. Our conclusions are the following: (1) The initial drying temperature during the processing of LWV tea affects its sensory quality. (2) R65 and R125 are not suitable temperatures for the initial drying of LWV. (3) R105 provided the highest sensory scores, which may be due to the content of amino acids, theanine, caffeine, and catechins. (4) The higher temperature of R125 significantly reduced the sensory profiles of LWV black tea during the first drying. (5) Drying temperature affected secondary metabolites, sesquiterpenoid and triterpenoid biosynthesis, glycerolipid metabolism, zeatin biosynthesis, phenylpropanoid biosynthesis, ABC transport, glutathione metabolism, and other pathways. This knowledge is suitable for black tea processing to produce an end product with the most optimum combination of flavor, biochemical profile, and volatile substance profiles.

## Figures and Tables

**Table 1:** Gradient elution conditions for detection of catechins content using liquid chromatography.

Time (min)	Flow Rate (mL/min)	Acetonitrile (%)	Water (%)
0.000	0.8	15	85
12.000	0.8	10	90
12.100	0.8	15	85
30.000	0.8	15	85

**Table 2:** Effect of processing temperature on catechin contents.

Sample Name	GA	GC	EGC	C	CAF	EC	EGCG	GCG	ECG	CG
Mixed Standard	4.17	5.24	8.20	10.67	12.56	18.68	19.221	22.552	31.28	38.6
R65	230.57 ± 7.59 a	93.775 ± 3.08 a	18.945 ± 0.83 a	34.1 ± 1.02 a	3650.65 ± 107.70 a	8.7 ± 0.26 a	274.32 ± 18.88 a	219.62 ± 5.21 a	28.25 ± 1.42 a	94.32 ± 8.70 a
R85	241.65 ± 18.68 a	97.15 ± 5.84 a	21.505 ± 1.17 ab	36.8 ± 7.03 a	3583.175 ± 259.10 a	14.6 ± 0.46 c	275.05 ± 47.29 a	228.22 ± 21.73 a	26.925 ± 2.05 a	118.1 ± 12.35 b
R105	257.05 ± 34.68 a	93.2 ± 10.74 a	23.56 ± 1.79 b	33.65 ± 3.56 a	3631 ± 238.44 a	15.805 ± 0.46 c	279.82 ± 30.37 a	213.45 ± 28.21 a	31.325 ± 5.19 a	110.15 ± 15.62 ab
R125	336.25 ± 12.31 b	85.125 ± 3.95 a	19.91 ± 0.85 a	32.8 ± 2.64 a	3376 ± 140.80 a	12.995 ± 1.29 b	219.72 ± 13.46 b	208.8 ± 1.87 a	27.075 ± 1.53 a	94.27 ± 4.19 a

GA, gallic acid; GC, gallocatechin; EGC, epigallocatechin; C, catechin; CAF, caffeine; EC, epicatechin; EGCG. epigallocatechin gallate; GCG, gallocatechin gallate; ECG, epicatechin gallate; CG, catechin gallate. R65, R85, R105, and R125 refer to processing temperatures 65 °C, 85 °C, 105 °C, and 125 °C, respectively. The values are means (*n* = 4) ± standard deviation. Different letters above mean significant difference at *p* < 0.05.

## Data Availability

All the data generated during this study are provided either in the main manuscript or as a Supplementary Material.

## Supplementary Materials

The following supporting information can be downloaded at: https://www.mdpi.com/article/10.3390/metabo15020074/s1, Figure S1. Scatter plot of the KEGG pathways in which the DAMs in R65 vs. R85 were significantly enriched, where R65 and R85 refer to the processing temperatures of 65 °C and 85 °C, respectively. Figure S2. Top differentially accumulated metabolites between R85 and R105, where R85 and R105 indicate the processing temperatures of 85 °C and 105 °C, respectively. Figure S3. Top differentially accumulated metabolites between R85 and R125, where R85 and R125 indicate the processing temperatures of 85 °C and 125 °C, respectively. Figure S4. Scatter plot of the KEGG pathways in which the DAMs in R85 vs. R125 were significantly enriched, where R85 and R125 refer to the processing temperatures of 85 °C and 125 °C, respectively. Figure S5. Top differentially accumulated metabolites between R105 and R125, where R105 and R125 indicate the processing temperatures of 105 °C and 125 °C, respectively. Figure S6. Scatter plot of the KEGG pathways in which the DAMs in R105 vs. R125 were significantly enriched, where R105 and R125 refer to the processing temperatures of 1055 °C and 125 °C, respectively. Table S1. List of differentially accumulated metabolites in LWV that are common in the four processing temperatures.

## References

[1] Pan S.-Y., Nie Q., Tai H.-C., Song X.-L., Tong Y.-F., Zhang L.-J.-F., Wu X.-W., Lin Z.-H., Zhang Y.-Y., Ye D.-Y. (2022). Tea and tea drinking: China’s outstanding contributions to the mankind. Chin. Med..

[2] Jason W. China’s Dominance in Tea Shows No Signs of Waning; Gravesend, Kent DA11 0DF, United Kingdom, 27.06.2024 2024. https://www.teaandcoffee.net/feature/34515/chinas-dominance-in-tea-shows-no-signs-of-waning/.

[3] Tang G.-Y., Meng X., Gan R.-Y., Zhao C.-N., Liu Q., Feng Y.-B., Li S., Wei X.-L., Atanasov A.G., Corke H. (2019). Health functions and related molecular mechanisms of tea components: An update review. Int. J. Mol. Sci..

[4] Aaqil M., Peng C., Kamal A., Nawaz T., Zhang F., Gong J. (2023). Tea Harvesting and Processing Techniques and Its Effect on Phytochemical Profile and Final Quality of Black Tea: A Review. Foods.

[5] Jolvis Pou K. (2016). Fermentation: The key step in the processing of black tea. J. Biosyst. Eng..

[6] Temple S.J., Temple C.M., Boxtel A.J.B.v., Clifford M.N. (2001). The effect of drying on black tea quality. J. Sci. Food Agric..

[7] Qu F., Zhu X., Ai Z., Ai Y., Qiu F., Ni D. (2019). Effect of different drying methods on the sensory quality and chemical components of black tea. Lwt.

[8] Teshome K. (2019). Effect of tea processing methods on biochemical composition and sensory quality of black tea (*Camellia sinensis* (L.) O. Kuntze): A review. J. Hortic. For..

[9] Kavish S., Botheju W.S., De Silva C.S. (2016). Impact of inlet drying temperature in endless chain pressure dryers on the quality characteristics of leafy type of tea produced using different leaf standards. OUSL J..

[10] Su S., Long P., Zhang Q., Wen M., Han Z., Zhou F., Ke J., Wan X., Ho C.-T., Zhang L. (2024). Chemical, sensory and biological variations of black tea under different drying temperatures. Food Chem..

[11] Polat A., Şat İ.G., Ilgaz Ş. (2018). Comparison of black tea volatiles depending on the grades and different drying temperatures. J. Food Process. Preserv..

[12] Shinde A., Das S., Datta A. (2013). Quality improvement of orthodox and CTC tea and performance enhancement by hybrid hot air–radio frequency (RF) dryer. J. Food Eng..

[13] Duan D., Ma F., Zhao L., Yin Y., Zheng Y., Xu X., Sun Y., Xue Y. (2022). Variation law and prediction model to determine the moisture content in tea during hot air drying. J. Food Process Eng..

[14] (2018). Methodology for Sensory Evaluation of Tea.

[15] (2013). Tea. Determination of Water Extracts Content.

[16] (2013). Tea. Determination of Free Amino Acids Content.

[17] (2018). Determination of Total Polyphenols and Catechins Content in Tea.

[18] (2017). Determination of Theanine in Tea-Suing High Performance Liquid Chromatograpy.

[19] Kanehisa M. (2016). KEGG bioinformatics resource for plant genomics and metabolomics. Plant Bioinform. Methods Protoc..

[20] Arakawa K., Kono N., Yamada Y., Mori H., Tomita M. (2005). KEGG-based pathway visualization tool for complex omics data. Silico Biol..

[21] Liang S., Gao Y., Fu Y.-Q., Chen J.-X., Yin J.-F., Xu Y.-Q. (2022). Innovative technologies in tea-beverage processing for quality improvement. Curr. Opin. Food Sci..

[22] Pou K.J., Paul S.K., Malakar S. (2019). Industrial processing of CTC black tea. Caffeinated and Cocoa Based Beverages.

[23] Zhong Y.-F., Chang R., Chen S.-M., Luo H.-Y., Wang J., Zhang Y., Zhang L. (2022). Quality and characteristic components of different types of Tuo tea. J. Food Saf. Qual..

[24] Gramza-Michałowska A. (2014). Caffeine in tea camellia sinensis—Content, absorption, benefits and risks of consumption. J. Nutr. Health Aging.

[25] Liang Y., Xu Y. (2001). Effect of pH on cream particle formation and solids extraction yield of black tea. Food Chem..

[26] Wang X., Wan X., Hu S., Pan C. (2008). Study on the increase mechanism of the caffeine content during the fermentation of tea with microorganisms. Food Chem..

[27] Kong X., Xu W., Zhang K., Chen G., Zeng X. (2023). Effects of reaction temperature, pH and duration on conversion of tea catechins and formation of theaflavins and theasinensins. Food Biosci..

[28] Donlao N., Ogawa Y. (2019). The influence of processing conditions on catechin, caffeine and chlorophyll contents of green tea (*Camelia sinensis*) leaves and infusions. LWT.

[29] ElGamal R., Song C., Rayan A.M., Liu C., Al-Rejaie S., ElMasry G. (2023). Thermal degradation of bioactive compounds during drying process of horticultural and agronomic products: A comprehensive overview. Agronomy.

[30] Li L., Sheng X., Zan J., Yuan H., Zong X., Jiang Y. (2023). Monitoring the dynamic change of catechins in black tea drying by using near-infrared spectroscopy and chemometrics. J. Food Compos. Anal..

[31] Yin X., Huang J.a., Huang J., Wu W., Tong T., Liu S., Zhou L., Liu Z., Zhang S. (2022). Identification of volatile and odor-active compounds in Hunan black tea by SPME/GC-MS and multivariate analysis. LWT.

[32] Xie Y., Zheng J.Y., Hou Y.J., Li J.H., Liu W.H., Sun X., Huang Y.P. (2023). Hot-air full drying driven metabolome changes in white tea (*Camellia sinensis* L.). Int. J. Food Prop..

[33] Xu W., Wang X., Jia W., Wen B., Liao S., Zhao Y., Tang Q., Li K., Hua Y., Yang Y. (2023). Dynamic changes in the major chemical and volatile components during the “Ziyan” tea wine processing. LWT.

[34] Yao H., Su H., Ma J., Zheng J., He W., Wu C., Hou Z., Zhao R., Zhou Q. (2023). Widely targeted volatileomics analysis reveals the typical aroma formation of Xinyang black tea during fermentation. Food Res. Int..

[35] Lu M., Sheng C., Ke H., Li T., Liu Q., Zhang J., Li L., Wang Y., Ning J. (2024). Revealing the differences in aroma of black tea under different drying methods based on GC–MS, GC-O. Food Chem. X.

[36] Wang H.-j., Hua J.-j., Jiang Y.-w., Wang J.-j., Yuan H.-b. (2020). Effect of different heat transfer modes during secondary drying on quality components, color and taste of Congou black tea. Food Sci..

[37] Yang Z., Tang J., Xue L., Peng Y. Study on high efficiency black tea fermentation control system based on multiple regression. Proceedings of the 2021 2nd International Conference on Artificial Intelligence and Information Systems.

[38] Kumazawa K., Masuda H. (2001). Change in the flavor of black tea drink during heat processing. J. Agric. Food Chem..

[39] Kidist Teshome K.T., Adugna Debela A.D., Weyessa Garedew W.G. (2013). Effect of drying temperature and duration on biochemical composition and quality of black tea (*Camellia sinensis* L.) O. Kuntze at Wush Wush, south western Ethiopia. Asian J. Plant Sci..

