# Supplementary material for: Effect of Four Different Initial Drying Temperatures on Biochemical Profile and Volatilome of Black Tea
Source: Metabolites. 2025 Jan 25;15(2):74. doi: 10.3390/metabo15020074 (PMC11857630; doi:10.3390/metabo15020074)
Supplement: Supplementary file 1 [file metabolites-15-00074-s001.zip › Supplementary Figures.pptx]

## Slide 1
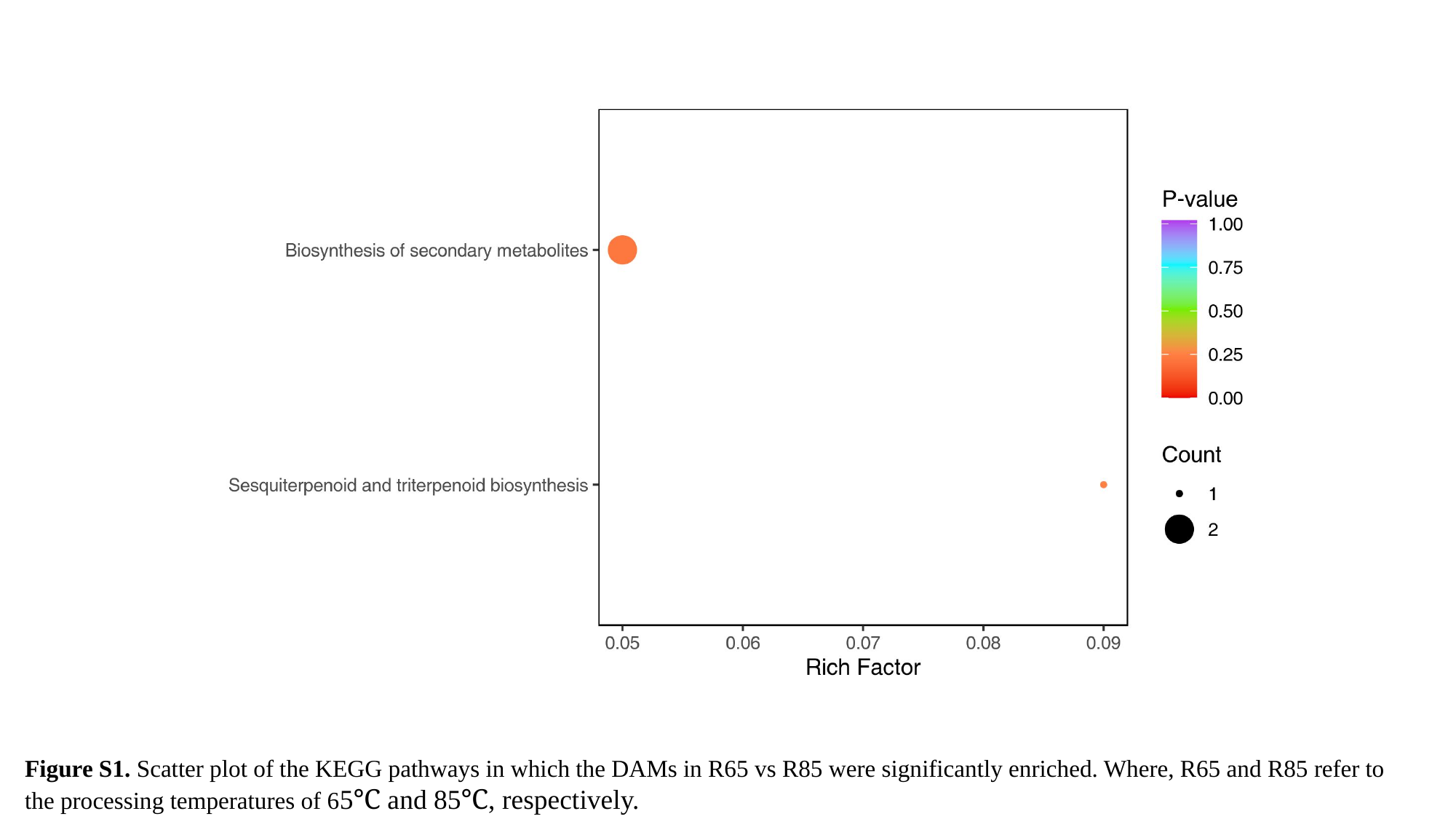

Figure S1. Scatter plot of the KEGG pathways in which the DAMs in R65 vs R85 were significantly enriched. Where, R65 and R85 refer to
the processing temperatures of 65℃ and 85℃, respectively.

## Slide 2
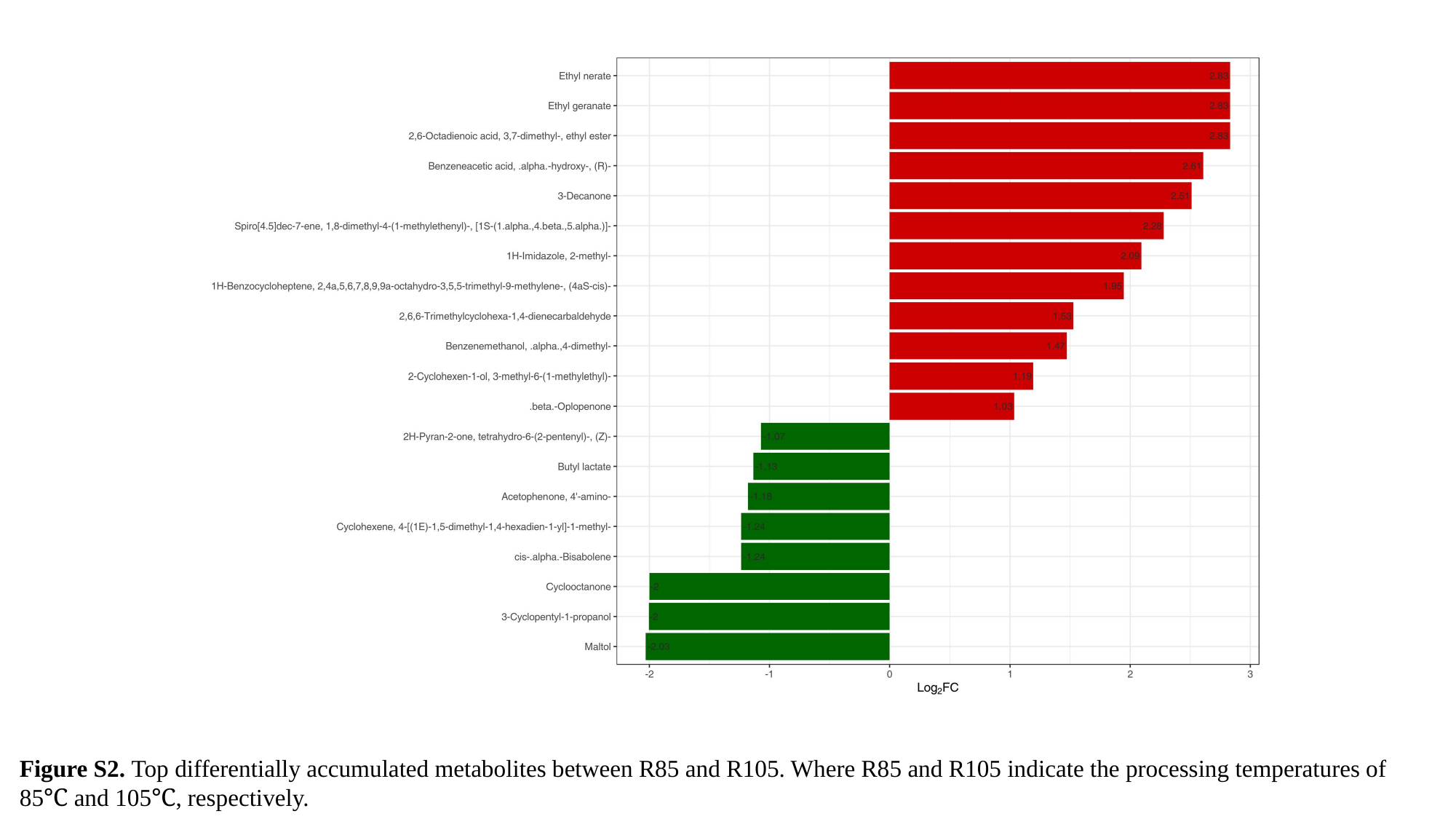

Figure S2. Top differentially accumulated metabolites between R85 and R105. Where R85 and R105 indicate the processing temperatures of
85℃ and 105℃, respectively.

## Slide 3
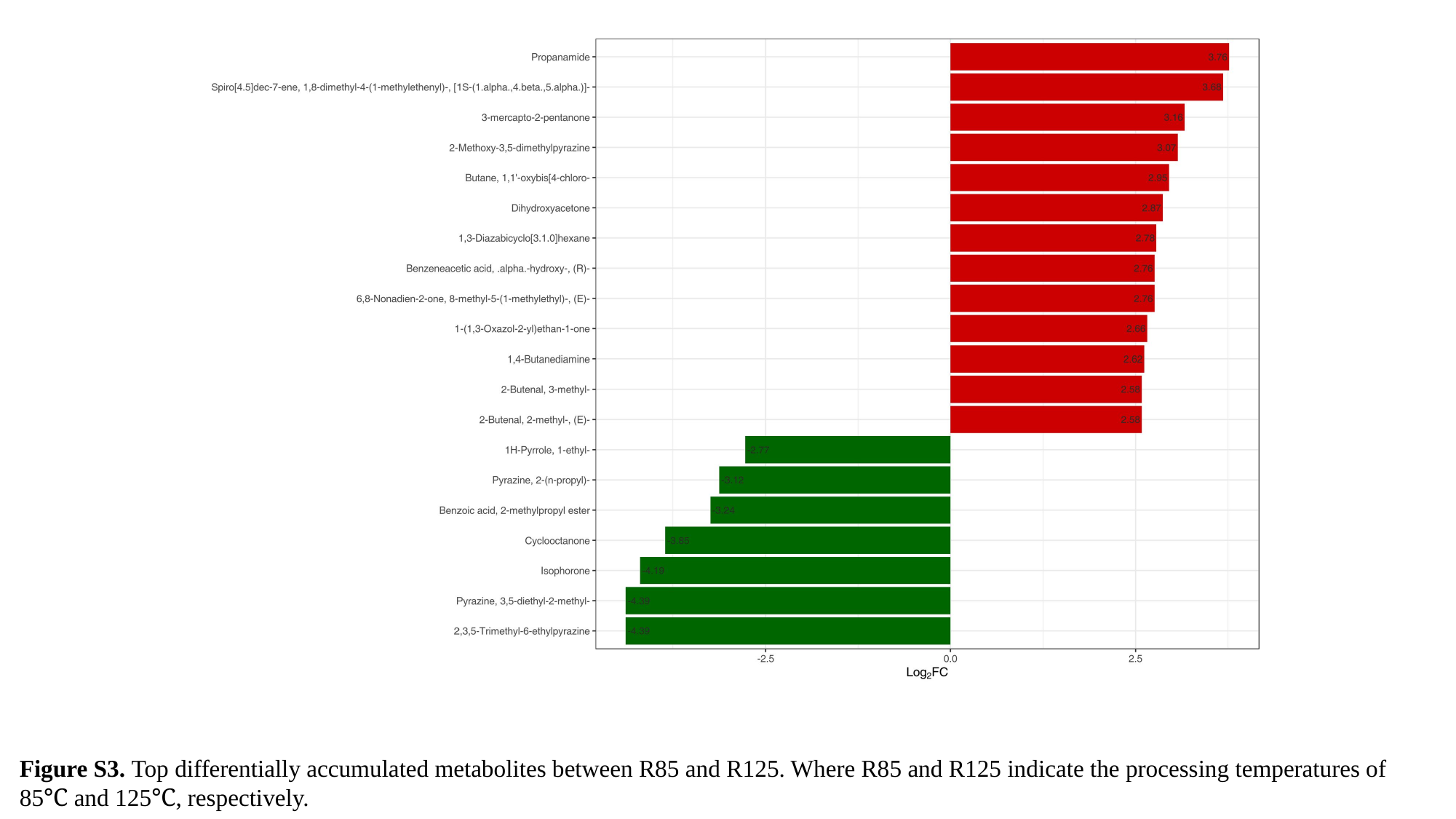

Figure S3. Top differentially accumulated metabolites between R85 and R125. Where R85 and R125 indicate the processing temperatures of
85℃ and 125℃, respectively.

## Slide 4
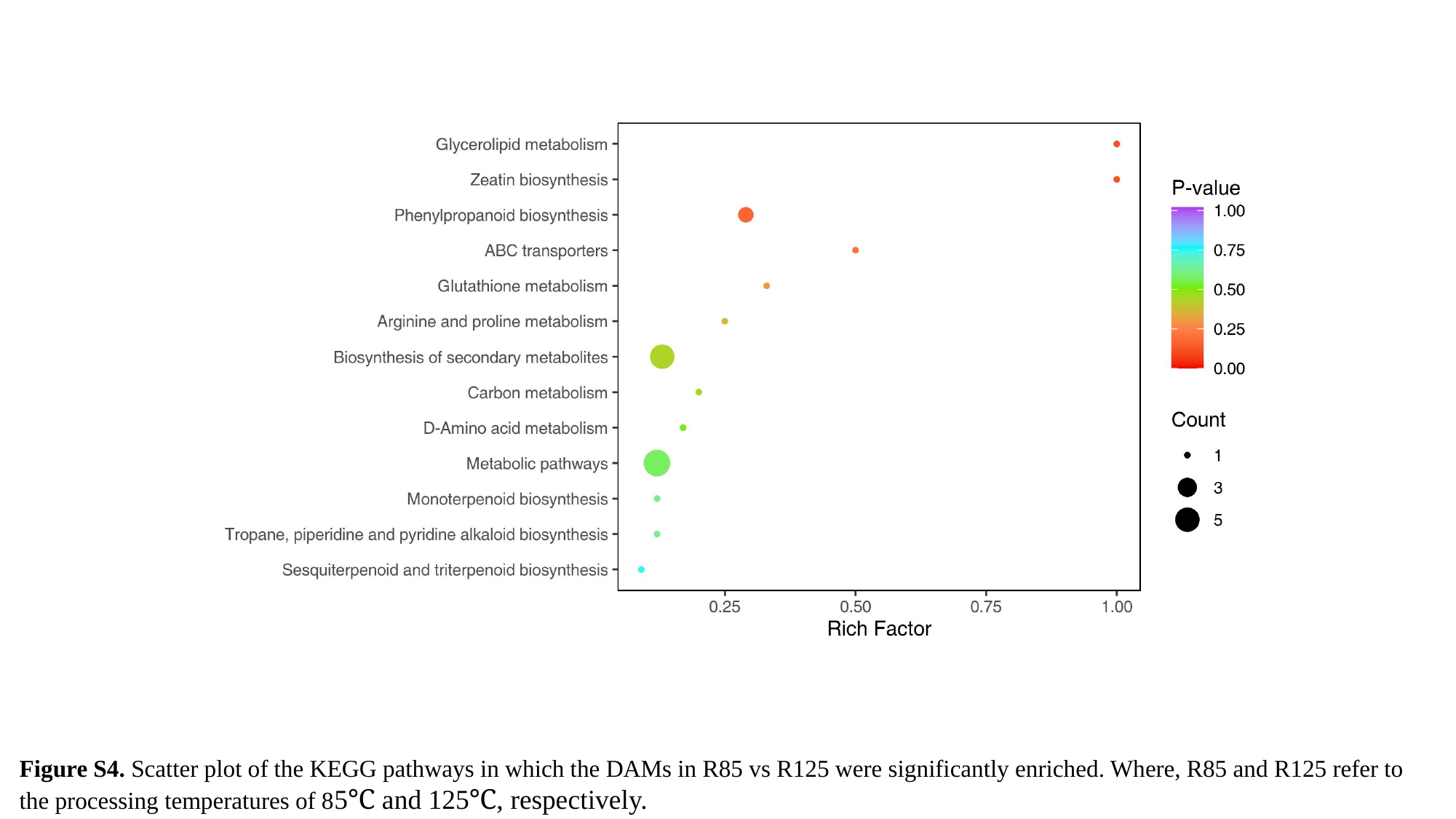

Figure S4. Scatter plot of the KEGG pathways in which the DAMs in R85 vs R125 were significantly enriched. Where, R85 and R125 refer to
the processing temperatures of 85℃ and 125℃, respectively.

## Slide 5
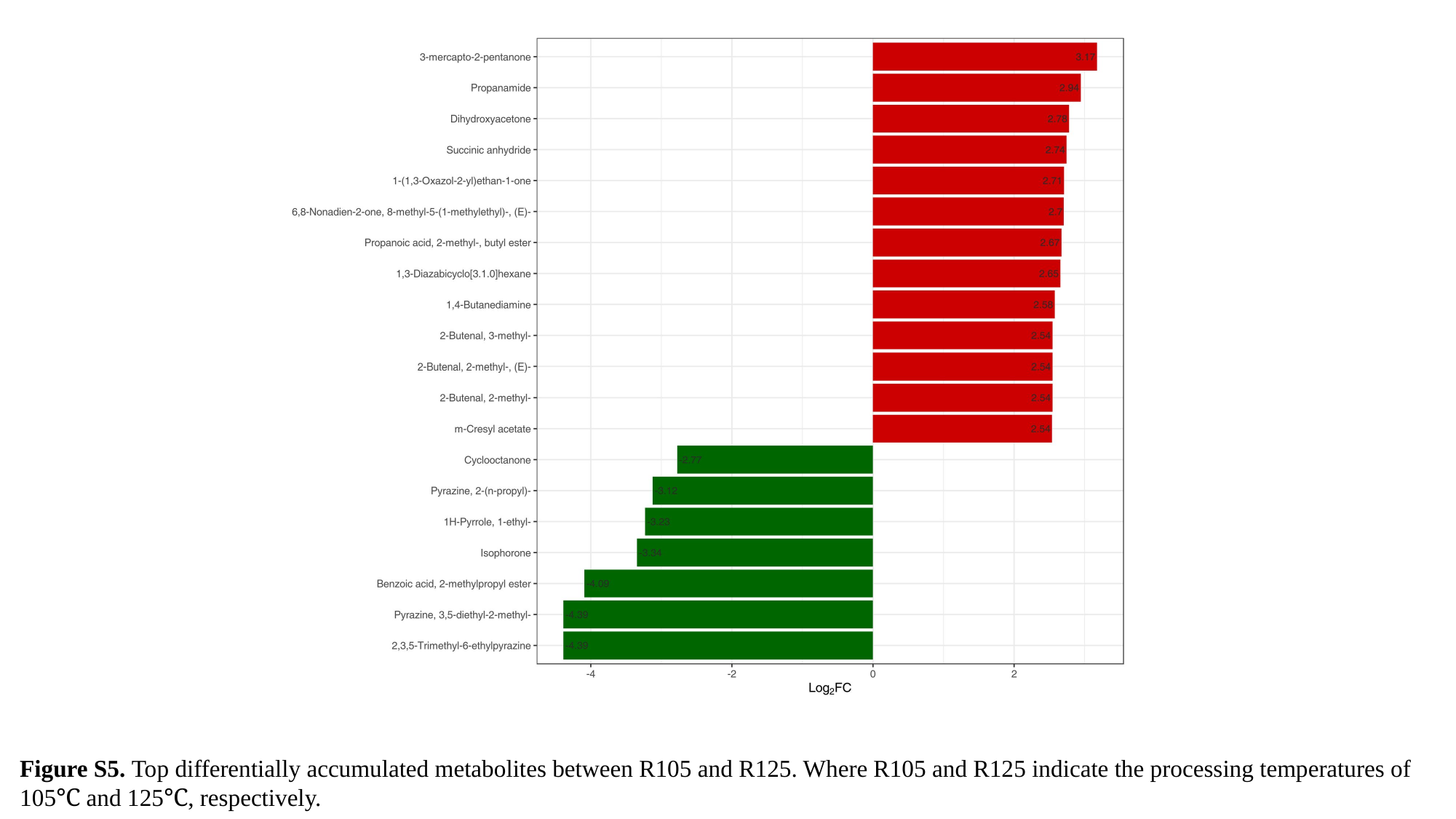

Figure S5. Top differentially accumulated metabolites between R105 and R125. Where R105 and R125 indicate the processing temperatures of
105℃ and 125℃, respectively.

## Slide 6
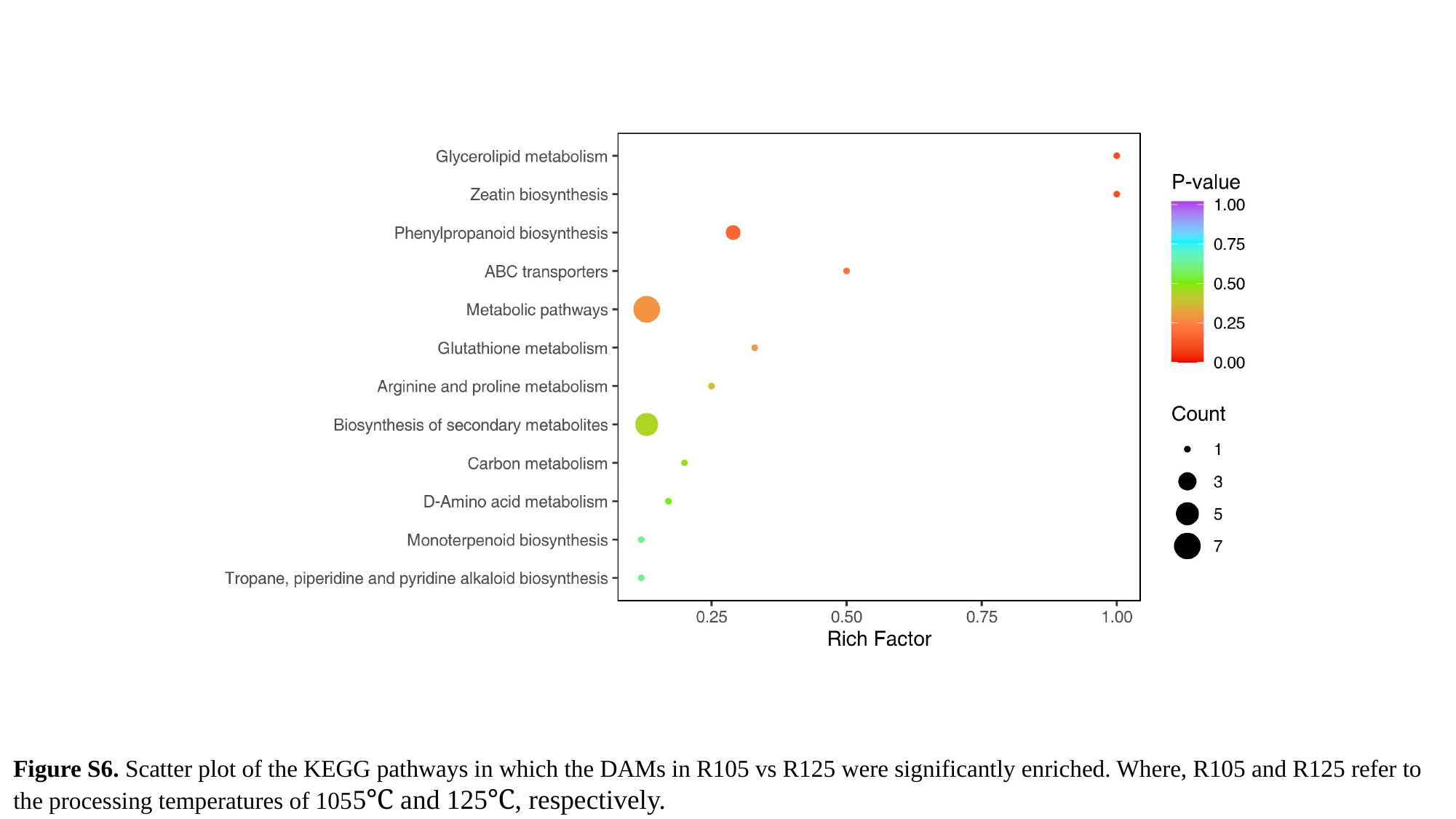

Figure S6. Scatter plot of the KEGG pathways in which the DAMs in R105 vs R125 were significantly enriched. Where, R105 and R125 refer to
the processing temperatures of 1055℃ and 125℃, respectively.
